# Incidental endometrial cancer diagnosis during pre-IVF workup: fertility-sparing treatment and embryo preservation

**DOI:** 10.1210/jcemcr/luag044

**Published:** 2026-03-24

**Authors:** Sonia Gayete-Lafuente, Umar Mian, Elizabeth Choong, Lara Guijarro-Baude, David Barad, Norbert Gleicher

**Affiliations:** Center for Human Reproduction (CHR), New York, NY 10021, USA; Foundation for Reproductive Medicine (FRM), New York, NY 10021, USA; Center for Human Reproduction (CHR), New York, NY 10021, USA; Center for Human Reproduction (CHR), New York, NY 10021, USA; Center for Human Reproduction (CHR), New York, NY 10021, USA; Department of Obstetrics and Gynecology, Hospital Universitari de Vic, Vic 08500, Spain; Center for Human Reproduction (CHR), New York, NY 10021, USA; Foundation for Reproductive Medicine (FRM), New York, NY 10021, USA; Center for Human Reproduction (CHR), New York, NY 10021, USA; Foundation for Reproductive Medicine (FRM), New York, NY 10021, USA; Department of Obstetrics and Gynecology, Medical University of Vienna, Vienna 1090, Austria; Laboratory of Synthetic Embryology, Rockefeller University, New York, NY 10065, USA

**Keywords:** endometrial cancer, nulliparous, IVF, fertility preservation, letrozole

## Abstract

We report the incidental diagnosis of endometrial cancer in a 42-year-old nulliparous woman undergoing routine fertility workup. During pre-in vitro fertilization (IVF) saline hysterosonography (HSN), a small (<1 cm) endometrial polyp at the fundus was identified and removed hysteroscopically. Initial histologic examination performed outside the United States demonstrated a grade 2 endometrioid carcinoma. The hysteroscopic pathology slides were subsequently reviewed in the United States, and the diagnosis was revised to grade 1, stage IA endometrioid carcinoma confined to the endometrium. Staging magnetic resonance imaging showed no myometrial invasion or metastatic disease, supporting a fertility-sparing management approach. Before initiating high-dose progestin therapy, the patient underwent fertility-preserving ovarian stimulation with a letrozole-based gonadotropin-releasing hormone antagonist protocol. This cycle yielded 4 embryos for cryopreservation. Had she proceeded with her planned IVF cycle before endometrial lesion was assessed, ovarian stimulation might have accelerated an undiagnosed estrogen-dependent malignancy. This case underscores the value of comprehensive pre-IVF diagnostic evaluation, through which incidentally detected early-stage endometrial carcinoma can be identified before ovarian stimulation and embryo transfer. It also highlights a potential role for fertility preservation in selected reproductive-aged patients and emphasizes the importance of guideline-based fertility-sparing management in estrogen-dependent gynecologic cancers.

## Introduction

Endometrial carcinoma is the most common gynecologic malignancy in high-income countries and its incidence among women aged 20-49 years has been rising [1 ]. Standard management with hysterectomy yields excellent 5-year survival rates but results in permanent infertility [2]. In reproductive-aged in vitro fertilization (IVF) patients, endometrial polyps are frequently detected on routine uterine cavity evaluation. Most are benign, but histologic assessment is essential to exclude atypical hyperplasia, atypia, or carcinoma [3 ]. We report a 42-year-old woman with infertility in whom a small endometrial polyp was detected on pre-IVF saline hysterosonography (HSN) and removed hysteroscopically. Histologic examination of the polyp revealed endometrioid carcinoma. Pathology review at a highly specialized oncology center confirmed a grade 1, stage IA tumor confined to the endometrium. These findings supported multidisciplinary fertility-sparing management plan that avoided hysterectomy. The patient first underwent fertility-preserving ovarian stimulation with oocyte retrieval and embryo cryopreservation, and then initiated high-dose progestin therapy.

## Case presentation

A 42-year-old nulliparous woman (G1P0) with a 7-year history of primary infertility presented to our center to pursue IVF. She had previously undergone 3 IVF cycles over 6 years at another clinic. Her menstrual cycles had recently become regular despite a history of polycystic ovary syndrome and a normal body mass index of 22.5 kg/m^2^, consistent with phenotype D under the Rotterdam criteria [4].

## Diagnostic assessment

During routine diagnostic workup, a saline HSN revealed a 6 × 9 mm sessile polyp in the left cornua of the uterine cavity. Because she was scheduled to travel to India, she was referred to a gynecologic surgeon there for hysteroscopic polypectomy. Histopathologic analysis of the resected polyp demonstrated a grade 2 endometrioid carcinoma with squamous differentiation arising in a hyperplastic endometrial polyp. The specimen showed crowded dysplastic glands with papillary architecture, nuclear atypia, squamoid edges, comedo-type necrosis, and solid areas involving approximately 30% of the tissue.

After she returned to the United States, hysteroscopic pathology slides were re-reviewed at a highly specialized oncology hospital. Upon review, the tumor was reclassified as a grade 1, stage IA endometrioid carcinoma confined to the endometrium.

Pelvic magnetic resonance imaging (MRI) confirmed the absence of myometrial invasion or extrauterine spread. These findings met National Comprehensive Cancer Network (NCCN) eligibility criteria for fertility-sparing management of endometrial cancer [5]. Such fertility-sparing treatment is increasingly applied and can be considered in carefully selected patients with grade 1, stage IA endometrioid carcinoma confined to the endometrium [6], although this decision should be individualized.

## Treatment

Desiring fertility preservation before definitive oncologic hormonal therapy, the patient was cleared by her oncologists for a rapid, modified ovarian stimulation protocol. She underwent a gonadotropin-releasing hormone (GnRH) antagonist regimen with ganirelix acetate 125 mcg subcutaneously once daily and the aromatase inhibitor letrozole 2.5 mg orally twice daily. Recombinant follicle-stimulating hormone (follitropin beta) 300 IU subcutaneously once daily was initiated on stimulation day 3. When the leading follicle reached a mean diameter of 22 mm on day 9, final oocyte maturation was triggered with leuprolide acetate 0.8 mg subcutaneously (GnRH agonist trigger per clinic protocol). Oocyte retrieval was performed 34 hours later.

The retrieval yielded 9 oocytes (7 metaphase II, 1 germinal vesicle, 1 atretic), 4 of which developed into cleavage-stage embryos (grade 8B4) and were cryopreserved.

In accordance with oncology guidance, conservative management of her endometrial carcinoma was initiated with high-dose megestrol acetate 40 mg orally 4 times daily. She subsequently enrolled in a clinical trial of targeted genomic therapy.

## Outcome and follow-up

The patient is undergoing endometrial biopsies every 10-12 weeks to monitor treatment response and reassess candidacy for pregnancy or additional embryo-banking cycles. Clearance to attempt pregnancy will be considered after 1 endometrial biopsy demonstrates complete histologic remission following 3 months of treatment.

## Discussion

This patient's cancer was detected incidentally during pre-IVF evaluation, highlighting the value of saline HSN with histologic sampling before embryo transfer. Most endometrial polyps in premenopausal women are benign, but a minority harbor premalignant or malignant changes. In a cohort of 150 women aged 29-52 years (mean age 42.1 ± 5.6 years), atypical hyperplasia or carcinoma was identified in 2.2% of endometrial polyps [3]. Pooled data estimate malignancy in about 1% for endometrial polyps in premenopausal women and 5% in postmenopausal women [7]. In an age-stratified series, malignant change was present in 2.5% of polyps in women aged 25-35 years and in roughly one-third of polyps in women older than 65 years [8]. These findings show malignancy risk is low, not absent, in younger women and rises sharply with age, so carcinoma cannot be excluded even in reproductive-aged patients such as our 42-year-old patient.

**Figure 1:**
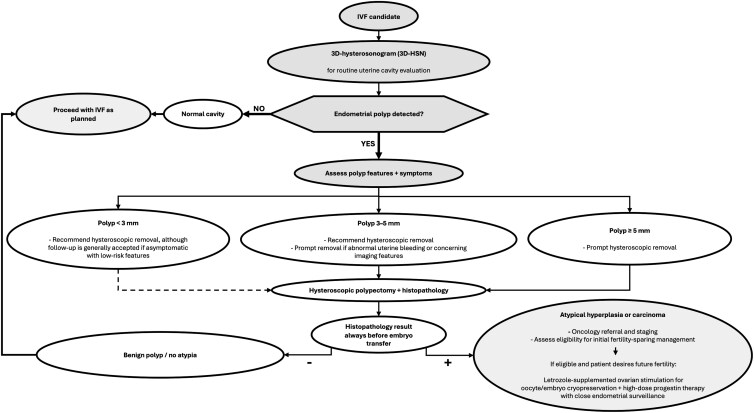
Suggested workflow for evaluation and management of endometrial polyps in patients pursuing in vitro fertilization (IVF). IVF candidates undergo routine uterine cavity evaluation with 3-dimensional saline HSN (3D-HSN). If the cavity is normal, IVF treatment proceeds as planned. When an endometrial polyp is detected on 3D-HSN, hysteroscopic removal is always recommended, and urgency and strength of indication is guided by polyp size and symptoms. For very small (<3 mm) asymptomatic polyps, although an expectant management with follow-up 3D-HSN is generally considered reasonable, a moderate recommendation for hysteroscopic removal may be considered given the low invasiveness of the procedure, particularly in the presence of abnormal uterine bleeding or concerning sonographic features. For polyps measuring 3-5 mm, hysteroscopic removal is strongly recommended, especially when alarming imaging features or symptoms are present. Polyps ≥5 mm are always routinely removed prior to embryo transfer. All polyps removed should be submitted for histopathologic assessment. Benign histology allows IVF treatment to proceed, whereas atypical hyperplasia or carcinoma warrants oncology referral, staging, and evaluation for fertility-sparing treatment. In appropriately selected patients desiring future fertility, fertility preservation may include letrozole-based GnRH-antagonist ovarian stimulation for oocyte or embryo cryopreservation followed by initiation of high-dose progestin therapy with close endometrial surveillance. Abbreviation: GnRH, gonadotropin-releasing hormone.

Hysteroscopic removal with histopathologic assessment remains the standard for diagnosis and helps optimize the uterine environment before embryo transfer. Although only polyps ≥5 mm have been robustly associated with reduced implantation rates, our experience supports recommending removal of all endometrial polyps detected before IVF to allow histopathologic evaluation. The strength and urgency of this recommendation vary with polyp size and clinical features (see Fig. 1): hysteroscopic removal of polyps ≥5 mm is always indicated; polyps 3-5 mm warrant prompt removal, particularly when abnormal bleeding or concerning imaging characteristics are present; and even very small (<3 mm) asymptomatic polyps are generally removed before embryo transfer, given the low procedural burden and small but clinically meaningful risk of atypia or carcinoma. In the present case, the 6 × 9 mm cornual polyp identified on HSN clearly met our institutional threshold for removal, and its excision ultimately enabled the definitive diagnosis of the underlying carcinoma.

Although hysteroscopy remains the diagnostic gold standard for direct visualization of focal intrauterine pathology, we do not advocate routine diagnostic hysteroscopy before fertility treatment in asymptomatic women with a normal-appearing uterine cavity on transvaginal ultrasound, due to lack of live birth benefit, but invasiveness and cost. Instead, our institutional policy is to use 3-dimensional saline HSN (3D-HSN) for uterine cavity evaluation before a first embryo transfer or transfer after delivery, miscarriage, or dilation and curettage (D&C), and whenever an intrauterine lesion (eg, polyp or fibroid) is suspected on ultrasound. Although both procedures are minimally invasive, hysteroscopy causes more discomfort than HSN [9]. In the United States, costs for elective HSN are about $400-$1400 per procedure, with an average charge around $960 [10]. When ordered for infertility, abnormal uterine bleeding, or suspected intrauterine pathology, HSN is often insurance-covered and therefore also more cost-efficient than routine diagnostic hysteroscopy. Comparatively, Medicare price estimates for outpatient hysteroscopic biopsy/polypectomy suggest total costs of approximately US$2000-US$4800 [11].

While malignancy or atypia occurs in only 1-2% of premenopausal endometrial polyps—necessitating roughly 50-100 polypectomies to detect 1 abnormal lesion—this case underscores the clinical importance of early identification in IVF candidates, where findings may meaningfully alter both oncologic and reproductive decision-making.

Fertility-sparing treatment is an increasingly accepted option for women with very early-stage endometrial cancer, particularly a grade 1 endometrioid histology. Recent studies report complete response rates from 66.7-79.7% in appropriately selected patients undergoing conservative management with progestins [2, 6, 12]. In this case, expert pathology review at a comprehensive cancer center reclassified the tumor from grade 2 to grade 1, and staging MRI confirmed disease confined to the endometrium (stage IA). Together, these findings met 2025 NCCN eligibility criteria for fertility-sparing management of endometrial cancer, avoiding hysterectomy [5]. Following confirmation of our patient's eligibility, she began fertility-sparing treatment with high-dose megestrol acetate, a synthetic progestin that suppresses endometrial proliferation by inducing stromal decidualization and exerting anti-estrogenic effects [13].

Because progestin therapy requires suppression of endogenous estrogen, fertility preservation is an important consideration for reproductive-aged women pursuing conservative management. In this patient, advanced reproductive age and the need to delay pregnancy until oncologic remission prompted rapid fertility preservation prior to initiating hormonal therapy. One IVF cycle with embryo cryopreservation was completed to safeguard reproductive potential, with conception planned only after oncologic remission and clearance. Although fertility preservation entails substantial costs, decision-analytic studies suggest that offering oocyte cryopreservation to women undergoing gonadotoxic or fertility-modifying cancer treatments can be cost-effective and a reasonable option in selected cases [14].

Ovarian stimulation for embryo cryopreservation in this patient was performed using a letrozole-based GnRH-antagonist protocol, an aromatase inhibitor strategy commonly used to limit estrogen exposure in estrogen-dependent tumors [15, 16]. Letrozole, a third-generation aromatase inhibitor, reduces peripheral estrogen synthesis without impairing hypothalamic-pituitary function, limiting theoretical tumor stimulation during controlled ovarian hyperstimulation (COS). Clinical studies demonstrate that letrozole-based COS reduces peak estradiol levels by ∼70% (mean peak estradiol ∼400 pg/mL or 1487 pmol/L) without compromising oocyte yield or embryo quality [16, 17]. At the end of stimulation, a GnRH agonist trigger was used for final follicular maturation to further minimize estrogen exposure before progestin therapy, consistent with international guidelines [18, 19].

Posttreatment surveillance includes endometrial biopsies every 10-12 weeks, with at least 1 negative biopsy after a minimum of 3 months of therapy required before pregnancy attempts, balancing reproductive goals with oncologic safety [20]. Published data show complete remission rates of approximately 70-84%, relapse rates of 13-27% and a posttreatment childbearing rate of about 20%, supporting fertility-sparing treatment as an effective, though not definitive, alternative to immediate radical surgery in selected patients [2, 6]. Pregnancy following fertility-sparing management of endometrial cancer is considered safe once oncologic criteria for remission are met, although close surveillance during gestation is recommended. A large multicenter cohort reported 62% live birth among women conceiving after complete remission following conservative treatment for endometrial cancer or atypical hyperplasia, with no increase in obstetric complications attributable to prior malignancy or its treatment [21]. Similarly, a large case series did not show unfavorable obstetric outcomes among women who conceived after conservative management of early-stage disease [22].

Incidental diagnosis of an early-stage endometrial carcinoma in this IVF candidate prompted a rapid shift to integrated fertility-preserving oncologic care. This case highlights the value of a structured workflow for evaluating endometrial polyps in IVF patients and identifying those eligible for fertility-sparing management, as summarized in Fig. 1. It emphasizes the importance of multidisciplinary collaboration in counseling women about oncologic safety, fertility preservation, and treatment timing, and shows how evolving fertility-sparing strategies can be tailored to individual patients [13].

## Learning points

- Early-stage endometrial carcinoma can be incidentally identified during pre-IVF uterine cavity evaluation (eg, saline HSN, ideally 3D-HSN), so abnormal findings (eg, endometrial polyps) should prompt structured assessment of size, location, symptoms, and imaging features, with hysteroscopic removal and histologic examination when indicated.
- Letrozole-based GnRH-antagonist controlled ovarian stimulation is a guideline-supported strategy for oocyte and embryo banking in women with estrogen-dependent malignancies and should be considered before initiating oncologic treatment in patients who desire future fertility.
- In carefully selected reproductive-aged patients with early-stage, low-grade endometrial carcinoma who desire future pregnancy, fertility-sparing progestin therapy with close oncologic surveillance can be a reasonable alternative to immediate radical surgery. This approach is best implemented within multidisciplinary care frameworks.

## Data Availability

Data sharing is not applicable to this article, as no datasets were generated or analyzed during the current study.
